# Biological and clinical factors predicting sperm retrieval success in men with non-obstructive azoospermia

**DOI:** 10.1530/RAF-26-0020

**Published:** 2026-09-30

**Authors:** Saffet Ozturk

**Affiliations:** Department of Histology and Embryology, Akdeniz University School of Medicine, Antalya, Türkiye

**Keywords:** sperm retrieval prediction, sperm generation, male infertility, non-obstructive azoospermia

## Abstract

**Abstract:**

Non-obstructive azoospermia (NOA) represents the most severe form of male infertility, characterized by impaired spermatogenesis and consequent failure to produce sufficient sperm. Accordingly, the identification of reliable predictors of testicular sperm presence has become a central focus of contemporary research. In parallel, considerable effort has been directed toward the development of strategies to generate sperm cells. Accumulating evidence indicates that histopathological findings, physical examination parameters, and patient demographics may contribute to the prediction of sperm retrieval outcomes. In addition, circulating reproductive hormone levels, including testosterone, as well as proteomic and transcriptomic profiles derived from semen and testicular tissues, have demonstrated potential utility in predicting success during testicular sperm retrieval. Emerging approaches aimed at sperm generation – such as the application of spermatogonial stem cells, induced pluripotent stem cells, and pharmacological interventions including aromatase inhibitors, estrogen modulators, and hormonal therapies – have also been increasingly investigated. Collectively, these advances suggest that both the prediction of sperm retrieval and the development of sperm production strategies in NOA are evolving rapidly, with promising implications for future clinical practice.

**Lay summary:**

Non-obstructive azoospermia (NOA) is a severe form of male infertility in which the testes produce little to no sperm. Determining whether sperm can be retrieved from the testes and whether sperm production can be restored are, therefore, critical clinical challenges. Recent evidence indicates that testicular histopathology, physical examination findings, patient characteristics, and circulating hormone levels, together with advanced molecular analyses, can aid in predicting the likelihood of successful sperm retrieval. In parallel, emerging approaches, including stem cell-based strategies and hormonal therapies, are being investigated to promote sperm production. Overall, advances in both prediction and therapeutic development suggest that achieving sperm retrieval or generation in men with NOA may be more feasible than previously anticipated.

## Introduction

Infertility affects approximately 15% of couples, with male-factor infertility accounting for nearly 30% of cases (Awonuga *et al.* 2025). Among infertile men, approximately 10–15% are diagnosed with azoospermia, defined as the absence of sperm in the ejaculate, confirmed by microscopic evaluation of at least two independently collected, centrifuged semen samples (Schlegel *et al.* 2021*a*,*b*). Azoospermia is broadly classified into two categories: obstructive (OA) and non-obstructive (NOA), accounting for approximately 40 and 60% of cases, respectively. OA is characterized by physical blockage of the spermatic ducts, most commonly due to scarring or prior surgical interventions. NOA has been reported in approximately 10% of infertile men and in about 1% of the general male population (Tao 2022). Its etiology is predominantly attributed to pre-testicular and testicular factors (Table 1). The primary etiology of pre-testicular factors is endocrine disruption, manifesting as impaired hypothalamic–pituitary–gonadal axis function, which in turn causes secondary testicular dysfunction (Fraietta *et al.* 2013). The cases with hypogonadotropic hypogonadism may benefit from exogenous gonadotropin therapy, including follicle-stimulating hormone (FSH) and human chorionic gonadotropin (hCG), to induce spermatogenesis (Kumar 2013). Current guidelines from the European Association of Urology (EAU) and the American Urological Association (AUA)/American Society for Reproductive Medicine (ASRM) do not recommend hormonal therapy for men with NOA, except in cases where there is clear evidence that such treatment effectively restores spermatogenesis (Salonia *et al.* 2021, Schlegel *et al.* 2021*a*,*b*).

**Table 1:** Etiological factors of non-obstructive azoospermia (NOA), underlying pathogenic mechanisms, and corresponding therapeutic approaches.

Etiological factors	Representative causes	Underlying mechanism(s)	Therapeutic approaches
Genetic abnormalities	Klinefelter syndrome Y chromosome microdeletions Monogenic defects	Meiotic arrest Sertoli cell dysfunction Germ cell depletion	Genetic counseling Gene-editing approaches
Endocrine disorders	Hypogonadotropic hypogonadism Pituitary disorders GnRH deficiency	Inadequate gonadotropin stimulation	hCG treatment Recombinant FSH treatment GnRH therapy Semen analysis mTESE/TESE/TESA
Cryptorchidism	Unilateral Bilateral	Testicular hyperthermia Impaired germ cell maturation Progressive germ cell loss	Early orchiopexy Hormonal therapy Semen analysis mTESE/TESE/TESA Fertility preservation strategies
Varicocele	Clinical Subclinical varicocele	Oxidative stress Increased scrotal temperature Hypoxia Disruption of the testicular microenvironment	Varicocelectomy Antioxidant therapy Semen analysis mTESE/cTESE/TESA
Testicular injury	Chemotherapy Radiotherapy Environmental toxins Occupational exposures Trauma	Direct germ cell depletion Stem cell loss Testicular tissue damage	Fertility preservation Sperm or testicular tissue cryopreservation SSC transplantation Semen analysis mTESE/cTESE/TESA
Idiopathic NOA	No identifiable cause	Genetic defects Epigenetic alterations Environmental factors	mTESE/TESE/TESA SSC-based therapies Artificial gametogenesis

FSH, follicle-stimulating hormone; GnRH, gonadotropin-releasing hormone; hCG, human chorionic gonadotropin; mTESE, microdissection testicular sperm extraction; NOA, non-obstructive azoospermia; SSC, spermatogonial stem cell.

Histopathologically, NOA encompasses a spectrum of testicular abnormalities, including Sertoli cell-only syndrome (SCOS), maturation arrest at early or late stages, and hypospermatogenesis (Mostafa *et al.* 2025). SCOS (also known as germ cell aplasia) is the most frequently observed histopathological pattern, followed by maturation arrest and hypospermatogenesis. SCOS is characterized by the absence of germ cells within the seminiferous tubules, which are populated exclusively by Sertoli cells. Notably, ultrastructural analyses have identified a limited number of spermatogonial cells in SCOS cases (Schleiermacher 1980, Zhao *et al.* 2020). Maturation arrest may occur at a specific stage or present as a generalized disruption of spermatogenesis, most commonly at the spermatogonial, primary spermatocyte, or round spermatid stages (Mostafa *et al.* 2025). Hypospermatogenesis is characterized by the presence of complete spermatogenesis, albeit at a quantitatively reduced level. A range of surgical methods are employed to retrieve sperm in patients with NOA, irrespective of the underlying histopathological pattern.

## Basic surgical methods for sperm retrieval in azoospermic patients

Although sperm retrieval rates approach 100% in men with OA, the success rate in patients with NOA ranges between 40 and 60% (Colpi *et al.* 2009, Verheyen *et al.* 2017). Epididymal or testicular sperm retrieval methods are utilized in azoospermic men in whom viable sperm are not recoverable from the ejaculate but remain potentially present within the male reproductive tract or testes. Epididymal sperm retrieval techniques, including percutaneous epididymal sperm aspiration (PESA) and microsurgical epididymal sperm aspiration (MESA), are widely employed due to their minimally invasive nature and high sperm retrieval success rates (Donoso *et al.* 2007, Leung *et al.* 2014). These techniques are commonly employed in men with OA, including those with vasectomy, congenital bilateral absence of the vas deferens, or epididymal obstruction, conditions in which spermatogenesis is generally preserved.

Testicular sperm retrieval techniques, including testicular sperm aspiration (TESA) or testicular sperm extraction (TESE), such as microdissection TESE (mTESE), are indicated in cases of NOA characterized by impaired or focal spermatogenesis (Schlegel 1999, Beliveau & Turek 2011, Esteves 2015). mTESE demonstrates superior sperm retrieval rates compared with conventional TESE (cTESE), TESA, or needle biopsy-based approaches in men with NOA (Arshad *et al.* 2020, Qi *et al.* 2021). The advantage of mTESE lies in its use of microscopic evaluation to identify dilated seminiferous tubules with a higher likelihood of active spermatogenesis, thereby minimizing unnecessary tissue excision and preserving testicular vascular integrity (Qi *et al.* 2021).

Aspiration-based techniques, such as TESA, involve percutaneous needle-dependent suction of seminiferous tubules, whereas extraction-based approaches, including TESE, involve open surgical biopsy with mechanical excision of testicular tissue for sperm retrieval (Esteves *et al.* 2013, Bernie *et al.* 2015). Although extraction techniques commonly contribute to higher sperm retrieval rates – particularly in men with NOA – they are associated with increased invasiveness and greater testicular tissue disruption. In these approaches, sperm cells – when present – can be successfully retrieved from discrete foci of spermatogenesis confined to limited regions of testicular parenchyma. Notably, the recovered sperm are routinely utilized for intracytoplasmic sperm injection (ICSI), irrespective of their qualitative characteristics or motility profiles.

mTESE has been widely recommended in the 2020 joint ASRM and AUA guidelines on male infertility as the preferred surgical approach for sperm retrieval in men with NOA (Schlegel *et al.* 2021*a*,*b*). This technique is associated with sperm retrieval rates of approximately 40–60%, whereas cTESE demonstrates lower rates, ranging from 15 to 45% (Deruyver *et al.* 2014). When compared with cTESE, the efficacy of mTESE is contingent upon the underlying histopathological pattern and the presence of either focal or diffuse spermatogenesis (Amer *et al.* 2000, Corona *et al.* 2019).

In men with SCOS, mTESE further enables the microsurgical identification of dilated seminiferous tubules, thereby enhancing sperm retrieval success rates. In the presence of maturation arrest, characterized by uniform impairment of spermatogenesis, mTESE confers only limited benefit. In cases of hypospermatogenesis, both approaches can be employed with comparable success. In addition, mTESE is superior to cTESE in mixed or heterogeneous histological phenotypes, particularly in detecting patchy spermatogenesis and decreasing sampling error. In the context of complications and tissue loss, mTESE is associated with a lower complication rate (just over 1%), reduced tissue excision, and improved preservation of testicular vascular integrity, whereas cTESE demonstrates higher complication rates (exceeding 3%) and greater tissue loss.

Trifocal mTESE has been shown to represent a more effective approach for predicting sperm retrieval success in patients with NOA, with a lower likelihood of retrieval-negative outcomes compared with conventional mTESE (Falcone *et al.* 2022). In more detail, the trifocal mTESE technique involves multiple equatorial and polar incisions, thereby enhancing access to a broader distribution of seminiferous tubules (Falcone *et al.* 2022, Barda *et al.* 2026). In contrast, conventional mTESE is characterized by a single and extensive tunical incision followed by microsurgical dissection (Colpi & Caroppo 2021). Although trifocal mTESE may be associated with prolonged operative time and an increased risk of vascular injury due to multiple tunical incisions, it has been reported to enhance the likelihood of successful sperm retrieval compared with the standard technique.

The reported success rates for mTESE, cTESE, and TEFNA are approximately 40–60, 15–45, and 10–30%, respectively (Yang *et al.* 2008, Bernie *et al.* 2015, Esteves 2022). Ghalayini *et al.* (2022) reported that in patients with NOA, a history of successful mTESE markedly increases the possibility of sperm retrieval in subsequent attempts, with approximately 10-, 6-, and 16-fold higher success rates observed in the second, third, and fourth procedures, respectively (Ghalayini *et al.* 2022). Preoperative total and free testosterone concentrations, smoking status, and a prior history of mTESE have been identified as significant predictors of mTESE outcomes (Al-Zubi *et al.* 2025). Importantly, infertile men harboring complete deletions in the *AZFa* and/or *AZFb* regions of the Y chromosome have virtually no likelihood of successful sperm retrieval through mTESE (Arshad *et al.* 2020).

As all sperm retrieval techniques are inherently invasive, accurate prediction of sperm retrieval outcomes is crucial for optimizing patient counseling and clinical decision-making. TESE procedures may be associated with adverse effects; therefore, accurate prediction of TESE outcomes may help minimize unnecessary interventions and reduce procedure-related morbidity (Zarezadeh *et al.* 2021). The following sections will discuss potential approaches for predicting sperm retrieval in men with NOA prior to surgical intervention, as well as the underlying potential for spermatogenesis in these patients.

## Sperm prediction in NOA patients

Accurate prediction of sperm presence in patients with NOA facilitates targeted sperm retrieval and may reduce the risk of complications associated with invasive surgical procedures. A substantial body of research has demonstrated that analyses of demographic characteristics, testicular volume, histopathological findings, hormonal profiles, and seminal plasma parameters may be valuable for predicting sperm recovery prior to TESE or other surgical interventions (Fig. 1).

**Figure 1:**
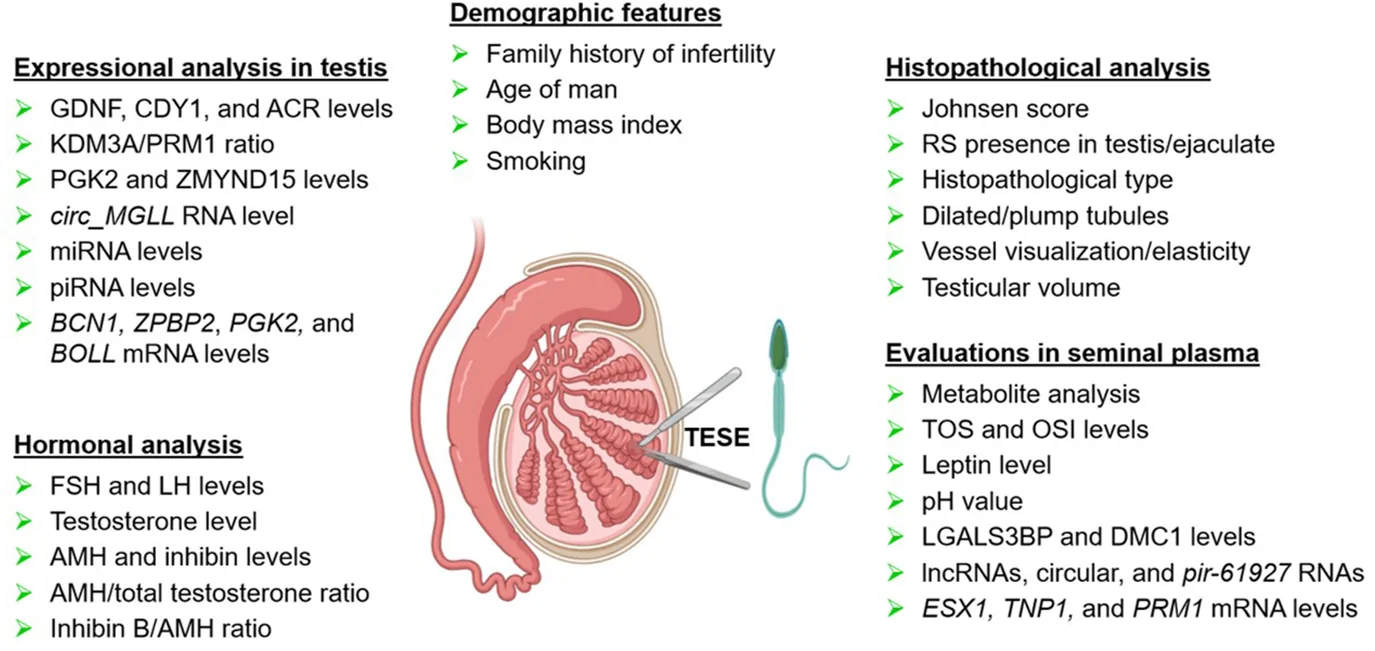
Basic features are used to predict the presence of sperm in NOA patients using the mTESE procedure. Various demographic characteristics, hormonal and other factor levels in serum and seminal plasma, expression analysis in testicular samples, and histopathological evaluations contribute to predicting sperm presence in these patients. The full names of all abbreviations are provided in the main text. This figure was created using the BioRender program (BioRender, Toronto, Canada).

### Demographic and clinical characteristics

#### Male age

Paternal age appears to influence sperm retrieval success in patients with NOA. Multivariable logistic regression analysis demonstrated a positive association between increasing paternal age and successful sperm retrieval in patients with NOA undergoing TESE (Cissen *et al.* 2016*a*) and mTESE (Amer *et al.* 2019). A recent study further reported a higher sperm retrieval success rate among older men undergoing TESE procedures, with a mean age of 34 years in the successful retrieval group compared with 33 years in the unsuccessful group (Ceyhan *et al.* 2025). The observed positive association between male age and sperm retrieval rate may be attributable to the easier identification of sperm-containing seminiferous tubules, despite the well-documented age-related decline in spermatogenesis.

Despite the aforementioned reports demonstrating a positive association between older paternal age and sperm retrieval outcomes, advancing paternal age may adversely affect spermatogenic activity and compromise the testicular microenvironment (Sharma *et al.* 2015). Some studies have reported a marked decline in sperm retrieval rates following mTESE with increasing age, particularly in men over 40 years, compared with younger cohorts. In advanced age, increased oxidative stress and accumulation of DNA damage within spermatogenic cells may impair spermatogonial stem cell renewal (Paul & Robaire 2013), thereby reducing the likelihood of focal active spermatogenesis as detected by mTESE. In addition, age-related alterations in epigenetic modifications such as DNA methylation patterns and telomere attrition within spermatogenic cells may compromise testicular regenerative capacity and impair spermatogenic activity in older men with NOA (Kaltsas 2025). Age-related partial alterations in testicular histopathological status such as maturation arrest and SCOS phenotypes may represent an additional factor contributing to the reduced likelihood of successful sperm retrieval. Collectively, paternal aging appears to represent a secondary clinical determinant associated with histopathological alterations and impaired spermatogenic activity, thereby influencing both the probability of successful sperm retrieval and even sperm quality.

#### Body mass index and smoking status

Among NOA patients with a history of bilateral cryptorchidism, those who achieved successful sperm retrieval following mTESE exhibited lower body mass index (BMI) values (Osaka *et al.* 2020). However, a more recently published meta-analysis demonstrated that men who underwent successful sperm retrieval exhibited significantly higher BMI values (Puia *et al.* 2023). Smoking has also been identified as an independent predictor of unfavorable sperm retrieval outcomes in patients diagnosed with NOA following TESE (Pavan-Jukic *et al.* 2020). Despite the conflicting evidence regarding the association between BMI and sperm retrieval success, lifestyle-related factors, including smoking status and dietary habits, appear to be significant predictors of sperm presence.

#### Testicular volume

In the context of clinical characteristics, the association between testicular volume and the predictive accuracy of sperm retrieval outcomes has been evaluated in several studies. Amer *et al.* (2019) reported that, among patients with NOA undergoing first-time mTESE, increased right testicular volume was positively associated with successful sperm retrieval. Among patients with NOA and a history of bilateral cryptorchidism, individuals with successful sperm retrieval following mTESE exhibited significantly greater mean testicular volume (Osaka *et al.* 2020). The authors stated that this parameter may serve as a predictor of successful sperm retrieval in patients with NOA. Similarly, median testicular volume was significantly greater in the sperm retrieval-positive group than in the sperm retrieval failure group (Saber-Khalaf *et al.* 2022). In the cohort of patients diagnosed with NOA, TESE demonstrated higher sperm retrieval success rates, which were positively correlated with increased testicular volume (Ceyhan *et al.* 2025, Zhou *et al.* 2025).

In contrast to the aforementioned studies, Zhang *et al.* (2020) demonstrated in men diagnosed with NOA that testicular volumes ≤5 mL exhibited higher sperm retrieval rates than those with testicular volumes of 5–10 or ≥10 mL (Zhang *et al.* 2020). Consistently, a recent study demonstrated that machine learning-based models contributed to the prediction of outcomes in patients with NOA, revealing a negative correlation with mean testicular volume (Xiao *et al.* 2025). These findings may be more reasonably interpreted as suggesting that an atrophic testis could facilitate the identification of focal areas of preserved spermatogenesis, rather than indicating an inherent biological advantage associated with smaller testicular size.

Overall, although testicular volume contributes to predicting sperm presence, the site of testicular sampling may also influence the success of TESE. Nevertheless, further mechanistic studies are warranted to elucidate the precise relationship between testicular volume and preserved spermatogenic activity.

### Imaging techniques and surgical factors

One of the key imaging modalities in the evaluation of NOA is scrotal ultrasonography, which allows for assessment of testicular volume, parenchymal echotexture, intratesticular lesions, and the presence of coexisting varicocele (Arshad *et al.* 2020, Qi *et al.* 2021). These parameters may provide valuable information regarding the extent of residual spermatogenesis. Color Doppler ultrasonography further enhances evaluation by assessing intratesticular perfusion, as vascular distribution appears to correlate with regions of active spermatogenesis, thereby guiding the optimal surgical target site for sperm retrieval (Schurich *et al.* 2009, Garolla *et al.* 2021). More recently, advanced imaging techniques – including contrast-enhanced ultrasonography, elastography, and testicular spectroscopy – have been proposed as tools to more precisely identify seminiferous tubules with residual sperm production, particularly in men undergoing mTESE (Celik *et al.* 2020).

Beyond surgical technique, several procedural and operator-dependent variables influence sperm retrieval success, including surgeon expertise, duration of microscopic evaluation, choice of exploration strategy (bilateral vs unilateral), and the efficiency of embryology laboratory assessment (Punjani *et al.* 2021). It is important to note that increasing the extent of microscopic dissection may enhance the detection of rare foci of spermatogenesis, particularly in men with NOA with histopathological patterns such as SCOS or maturation arrest (Arshad *et al.* 2020). In cases of failure in sperm retrieval in unilateral dissection, bilateral exploration should be an option to achieve a successful outcome. This observation underscores the critical importance of surgical expertise and intraoperative decision-making.

Accordingly, imaging and surgical parameters serve as complementary determinants in predicting sperm retrieval in men with NOA. Advances in functional ultrasonography and microsurgical mapping, coupled with increasing surgical expertise, may enhance the likelihood of identifying sperm within focal regions of the testes. Furthermore, the selection of imaging modalities and operative strategies appears to be increasingly individualized.

### Histopathological status

Histopathological evaluation of TESE specimens obtained from patients with NOA is conducted to determine the presence of spermatogenic activity and to characterize the underlying testicular histopathology. Several studies have suggested that these histopathological assessments may provide clinically relevant insights regarding the likelihood of successful sperm retrieval. Histopathological parameters, particularly the presence of testicular spermatids and the Johnsen score (optimal cut-off value: 4.8; odds ratio ≥6), were identified as independent predictors of successful sperm retrieval, with predictive accuracies of 77 and 71%, respectively (Ezeh *et al.* 1999). Similarly, successful sperm retrieval in men with NOA has been shown to be associated with a Johnsen score cut-off value of 5 (Rohan *et al.* 2021). Conversely, Cetinkaya *et al.* (2015) reported that a Johnsen score cut-off value of 2 provided the optimal predictive performance for sperm retrieval during mTESE, with a sensitivity of 71.2% and a specificity of 74.7%, identifying it as an independent predictor of sperm presence (Cetinkaya *et al.* 2015). A recent study employing multivariate logistic regression analysis demonstrated that higher Johnsen scores in testicular tissue specimens from patients with idiopathic NOA were positively associated with successful sperm retrieval during mTESE procedures (Shi *et al.* 2025). These findings suggest that a Johnsen score of ≥5, characterized by the presence of numerous spermatocytes in the absence of sperm or spermatids, may improve the predictive accuracy for sperm presence in TESE specimens. Nevertheless, because the Johnsen score is highly dependent on the site of testicular sampling, isolated seminiferous tubules located in different regions of the testis may still exhibit focal areas of preserved spermatogenesis.

The histological demonstration of complete spermatogenesis has been recognized as a robust prognostic marker for the presence of retrievable sperm. In other words, a stepwise decline in sperm retrieval rates has been observed in cases of hypospermatogenesis, maturation arrest, and SCOS, as consistently demonstrated by histopathological evaluation (Yang *et al.* 2008, Ghalayini *et al.* 2011, Caroppo *et al.* 2017). Indeed, hypospermatogenesis on histopathological examination is associated with higher rates of sperm retrieval, as well as improved clinical pregnancy, live birth, and overall spermatogenic outcomes (Kavoussi *et al.* 2020). As reported by Cito *et al.* (2018), the sperm retrieval rate was 24.1% in patients with maturation arrest, compared with 48.2% in those with hypospermatogenesis (Cito *et al.* 2018). Although no sperm retrieval was achieved in patients with SCOS in this study (Cito *et al.* 2018), successful sperm retrieval in SCOS has been documented in other reports (Gul *et al.* 2013, Modarresi *et al.* 2015). As a result, the histopathological pattern observed in patients with NOA appears to significantly influence the likelihood of successful sperm retrieval.

#### Intraoperative and post-procedure laboratory observations

From a seminiferous morphological perspective, in patients with NOA, seminiferous tubules displaying a dilated and plump appearance were found to contain sperm, indicative of active spermatogenesis (Smith *et al.* 2021). In contrast, seminiferous tubules that appeared non-dilated and translucent were devoid of sperm (Smith *et al.* 2021). In other words, the sperm retrieval rate progressively declined according to seminiferous tubule morphology, from dilated tubules (90%) to slightly enlarged tubules (47%) and ultimately to non-dilated tubules (7%) (Caroppo *et al.* 2019). A positive correlation was also identified between the presence of visible vessels within individual intratesticular segments and the likelihood of successful sperm retrieval from TESE biopsies obtained from the corresponding segments (Nowroozi *et al.* 2015). In addition to the histopathological status and morphological characteristics of seminiferous tubules, the proportions of pachytene spermatocytes, sperm, and Sertoli cells within the cell suspension may reflect the degree of spermatogenic status (Hessel *et al.* 2015). More detailed analyses demonstrated that the ratio of pachytene spermatocytes to sperm, together with the number of sperm per hundred Sertoli cells, could discriminate among complete spermatogenesis, hypospermatogenesis, and partial maturation arrest (Hessel *et al.* 2015). The integration of spermatogenic cell counts with seminiferous tubule morphology and testicular histopathology may contribute to improved prediction of sperm retrieval rates in patients with NOA. The combined assessment of these parameters (Fig. 1), particularly when analyzed using artificial intelligence approaches, may improve the predictive accuracy for the presence of sperm.

### Reproductive hormone levels

#### Follicle-stimulating and luteinizing hormones

Gonadotropins, including FSH and luteinizing hormone (LH), secreted by the adenohypophysis, play essential roles in the regulation and progression of spermatogenesis, ultimately facilitating the production of mature sperm (Bhattacharya *et al.* 2023, Esteves & Humaidan 2025). More specifically, FSH contributes to the proliferation of Sertoli cells during early postnatal development, thereby establishing the cellular framework necessary for subsequent spermatogenesis. Within the spermatogenic compartment, FSH has been shown to promote spermatogonial survival by modulating intrinsic apoptotic pathways, thereby contributing to the maintenance and restoration of the spermatogonial population (Maroto *et al.* 2025). In concert with testosterone, FSH facilitates the entry of spermatocytes into meiosis and supports their survival throughout meiotic progression. FSH also participates in the completion of meiosis, induces the differentiation of spermatocytes into round spermatids, and supports the process of spermiation (Ruwanpura *et al.* 2010). LH plays a central role in stimulating testosterone synthesis by Leydig cells, which are located in the interstitial (intertubular) compartment in close proximity to the testicular vasculature. Given the well-documented impairment of spermatogenic activity in patients with NOA, assessment of reproductive hormone profiles may provide a useful approach for predicting the likelihood of sperm retrieval in TESE samples (Fig. 1 and Table 2).

**Table 2:** Predictive significance of serum FSH and LH levels and their ratio for sperm retrieval outcomes in patients with NOA. The FSH/LH values are presented sequentially, with the first value corresponding to FSH and the second to LH.

Hormonal concentration (mIU/mL or IU/L)	Study method	Sperm retrieval success	Reference
FSH level			
9.35	Multivariate logistic regression	**↑**	Zhou *et al.* (2025)
>14.6	ROC	**↑**	Jahromi *et al.* (2020)
15.03	Univariate analysis	**↑**	Rohan *et al.* (2021)
>15.25	Multiple logistic regression	**↑**	Modarresi *et al.* (2015)
<16.05	ROC	**↑**	de Souza *et al.* (2003)
18.3	Multivariate logistic regression	**↑**	Nagata *et al.* (2005)
>19.4	ROC	**↑**	Chen *et al.* (2010)
16–26	Uni- and multi-variable binary logistic regression analyses	**↑**	Cissen *et al.* (2016*a*)
20.4	Uni- and multi-variate analyses	**↑**	Yucel *et al.* (2018)
LH level			
>24.8	Chi-squared or Fisher exact test	**↑**	Zhang *et al.* (2020)
12.20	Logistic regression and ROC	**↓**	Liu *et al.* (2020)
>15	Univariate analysis	**↓**	Yildirim *et al.* (2014)
Both FSH/LH levels			
25.97/9.9	Uni- and multi-variate analyses	**↑**	Osaka *et al.* (2020)
13/8	Uni- and multi-variate analyses	**↑**	Saber-Khalaf *et al.* (2022)
6.59/4.5	ROC	**↑**	Rachman *et al.* (2023)
≤12.92/≤6.07	Univariate logistic regression	**↑**	Ceyhan *et al.* (2025)
<22.29/<8.55	Uni- and multi-variate logistic regression	**↑**	Shi *et al.* (2025)

ROC, receiver operating characteristic; TESE, testicular sperm extraction; mTESE, microdissection TESE; FSH, follicle-stimulating hormone; LH, luteinizing hormone; NOA, non-obstructive azoospermia.

The literature demonstrates inconsistent findings regarding the predictive utility of serum FSH (de Souza *et al.* 2003, Nagata *et al.* 2005, Chen *et al.* 2010, Yildirim *et al.* 2014, Modarresi *et al.* 2015, Cissen *et al.* 2016*a*,*b*, Yucel *et al.* 2018, Jahromi *et al.* 2020, Liu *et al.* 2020, Zhang *et al.* 2020, Rohan *et al.* 2021, Zhou *et al.* 2025) and both FSH and LH (Osaka *et al.* 2020, Saber-Khalaf *et al.* 2022, Rachman *et al.* 2023, Ceyhan *et al.* 2025, Shi *et al.* 2025) levels for sperm retrieval success in men with NOA (Table 2). These parameters may hold clinical relevance for predicting the presence of sperm in selected cases. In this context, Tsujimura *et al.* (2005) created a mathematical formula *P* = (1 + exp (0.144 × patient age − 0.059 × serum FSH concentration − 1.310 × Johnsen score)) − 1 using serum FSH level as well as male age and Johnsen score (Tsujimura *et al.* 2005). Application of this formula to younger patients has been shown to enhance the accuracy of predicting sperm presence (Tsujimura *et al.* 2005). However, its accuracy across other NOA cohorts remains to be elucidated.

Collectively, these findings indicate that sustaining an appropriate balance of FSH and LH is essential for the regulation and maintenance of optimal spermatogenesis. The observed variability in FSH and LH concentrations (Table 2) may be attributable to differences in study methodologies, heterogeneity in histopathological findings, and interindividual variations associated with ethnicity and endocrine regulatory mechanisms. Further large-scale and well-standardized studies are warranted to establish a consensus regarding the FSH and LH concentration ranges most closely associated with sperm retrieval prediction.

#### Testosterone, anti-Müllerian hormone, and inhibin B

Testosterone has been shown to play a critical role in the differentiation and maintenance of Sertoli cell survival during early postnatal development (Ruwanpura *et al.* 2010). This hormone facilitates meiotic entry and supports spermatocyte survival through the regulation of both intrinsic and extrinsic apoptotic pathways (Maroto *et al.* 2025). Furthermore, testosterone plays an essential role in multiple stages of spermatogenesis, including meiotic completion, round spermatid differentiation, and spermiogenesis. Testosterone has been shown to promote the adhesion of round spermatids to Sertoli cells and to facilitate the subsequent process of spermiation (Ruwanpura *et al.* 2010, Smith & Walker 2014).

In a cohort of patients with NOA, total testosterone (TT) levels (optimal cut-off: 400 ng/dL) were identified as a potential predictor of sperm retrieval success during mTESE (Cetinkaya *et al.* 2015). Multivariable logistic regression analysis demonstrated an association between successful sperm retrieval and higher serum testosterone concentrations (10–20 nmol/L, approximately equal to 2.8–5.7 ng/mL) (Cissen *et al.* 2016*a*,*b*). Rohan *et al.* (2021) reported that successful sperm retrieval was associated with a testosterone cut-off value of 13 nmol/L, approximately equal to 3.8 ng/mL, in men with NOA undergoing mTESE (Rohan *et al.* 2021). In a cohort of patients with NOA and a history of bilateral cryptorchidism, serum testosterone levels were higher in the sperm retrieval-positive group (5 ng/mL) than in the sperm retrieval-negative group (4 ng/mL) (Saber-Khalaf *et al.* 2022). The presence of normal intratesticular testosterone levels may aid in predicting surgical sperm retrieval outcomes in patients with NOA who are unresponsive to hormonal therapy (Caroppo & Colpi 2023).

Higher serum testosterone levels (388.5 ng/dL, approximately equal to 3.8 ng/mL) were associated with an increased likelihood of successful sperm retrieval compared with the sperm retrieval-negative group in patients undergoing TESE (Ceyhan *et al.* 2025). Similarly, multivariate regression analysis demonstrated a positive association between serum testosterone levels and successful outcomes of TESE in patients with NOA (Zhou *et al.* 2025). Taken together, these studies suggest that serum testosterone concentrations in the range of approximately 2.8–5.7 ng/mL may serve as a reliable predictor of successful sperm retrieval in patients with NOA. Given the essential physiological roles of testosterone, its circulating levels are positively associated with the successful progression of spermatogenesis. Therefore, increasing intratesticular testosterone concentrations appears to be a potentially effective strategy for promoting spermatogenesis in NOA cases that exhibit reduced levels.

Anti-Müllerian hormone (AMH), also referred to as Müllerian-inhibiting substance, is a glycoprotein produced and secreted by Sertoli cells. Its primary physiological role is to mediate regression of the Müllerian ducts during embryonic development (An *et al.* 2023). However, the precise role of AMH in spermatogenic cells during spermatogenesis remains poorly understood. Pozzi *et al.* (2023) reported that serum AMH levels below 4 ng/mL may serve as a valuable predictor of successful sperm retrieval following mTESE in infertile men diagnosed with NOA. As an independent predictor, AMH levels were found to be inversely associated with successful sperm retrieval in patients with NOA (Zheng *et al.* 2024). Collectively, the limited number of studies demonstrated an inverse correlation between AMH concentration and sperm retrieval success.

The ratio of AMH to TT was identified as an independent predictor of sperm presence in patients with NOA (Alfano *et al.* 2017). Indeed, an AMH level < 4.62 ng/mL and an AMH/TT ratio <1.02 may serve as predictive thresholds for successful sperm retrieval in these patients. By integrating the concentrations of multiple hormones, Tsujimura *et al.* (2004) developed a predictive formula for predicting sperm presence. The formula was expressed as *P* = (1 + exp (5.201 − 0.048 × FSH − 0.449 × TT − 0.021 × inhibin B)) (−1)), incorporating serum FSH, TT, and inhibin B levels. In this study, a predicted probability threshold of approximately >16% was identified as the optimal cut-off for sperm retrieval prediction. As a result, incorporating multiple hormonal parameters within a single predictive model may enhance the accuracy of identifying patients with a higher likelihood of sperm retrieval during TESE procedures.

Inhibin B is a polypeptide hormone primarily produced by Sertoli cells and plays a critical role in the regulation of spermatogenesis and the inhibition of FSH secretion from the pituitary gland. Comparative analyses of serum inhibin B concentrations between patients with successful TESE outcomes (TESE-positive) and those with unsuccessful TESE outcomes (TESE-negative) demonstrated significantly higher inhibin B levels in the TESE-positive group (Ballesca *et al.* 2000). In a cohort of patients diagnosed with NOA, seminal inhibin B levels were reported to be significantly higher in the TESE-positive group than in the TESE-failed group (Nagata *et al.* 2005). This finding may be attributable to reduced FSH levels observed in TESE-positive patients diagnosed with NOA.

In patients with idiopathic NOA, serum inhibin B levels and the inhibin B/AMH ratio may serve as prognostic biomarkers for predicting sperm retrieval success during mTESE (Deng *et al.* 2023). Another recently published meta-analysis further demonstrated that men with NOA who achieved successful sperm retrieval exhibited significantly higher inhibin B levels (Puia *et al.* 2023). Machine learning-based models have also been shown to contribute to the prediction of sperm presence in patients with NOA, with mean inhibin B levels demonstrating a significant negative correlation within predictive analyses (Xiao *et al.* 2025). Despite conflicting findings regarding the precise relationship between inhibin B levels and sperm retrieval outcomes, higher inhibin B concentrations appear to be a potentially useful predictor of sperm presence. However, further studies are warranted to elucidate the underlying mechanistic relationship between inhibin B and spermatogenesis.

### Evaluation of seminal plasma

#### Biomolecular analysis

Seminal plasma, apart from sperm cells, contains a diverse array of biomolecules, including metabolites, proteins, and oxidant/antioxidant factors, which may serve as potential predictors of spermatogenic activity (Fig. 1).

Seminal plasma is known to contain a wide range of metabolites involved in bioenergetic pathways, membrane remodeling, and oxidative stress regulation, including carnitines, citrate, lactate, glycine, serine, tryptophan, and branched-chain amino acids, as well as lipid species such as phosphatidylserine, ceramides, diacylglycerols, and triglyceride-derived molecules. These metabolites are closely linked to sperm function, with multiple studies demonstrating significant associations with sperm concentration, motility, and morphology, reflecting their roles in energy metabolism, membrane integrity, and redox homeostasis during spermatogenesis (Qiao *et al.* 2017, Correnti *et al.* 2023). Accordingly, disruptions in amino acid biosynthetic pathways, carnitine depletion, and alterations in phospholipid architecture have been associated with emerging infertile phenotypes, varicocele-associated infertility, and idiopathic male infertility, highlighting their potential utility as noninvasive predictive biomarkers.

Seminal plasma proteomic profiling enables the identification of spermatogenesis-related biomarkers. Differential expression of proteins including testis-expressed 101 (TEX101), acrosomal vesicle protein 1 (ACRV1), extracellular matrix protein 1 (ECM1), sperm-associated antigen 11 (SPAG11), clusterin, prostaglandin D2 synthase (PTGDS), and galectin-3-binding protein (LGALS3BP) supports discrimination between OA and NOA, largely through their association with residual focal spermatogenesis and their utility in predicting sperm retrieval outcomes (Bieniek *et al.* 2016, Parvin *et al.* 2024*a*). TEX101 and ECM1 have been implicated in distinguishing testicular failure from obstructive etiologies within the male reproductive tract (Drabovich *et al.* 2013), whereas ACRV1 and SPAG11 are recognized as markers of epididymal maturation and sperm functional competence (Sullivan & Mieusset 2016). Clusterin provides diagnostic value as a marker of membrane remodeling, apoptotic stress responses, and impaired sperm maturation (Ibrahim *et al.* 2000).

As is well established, leptin is secreted by adipocytes and plays a central role in the long-term regulation of energy homeostasis. Seminal plasma leptin levels have been identified as a potential contributory biomarker for predicting sperm retrieval outcomes in patients undergoing mTESE (Amer *et al.* 2023). The lectin galactoside-binding soluble 3-binding protein (LGALS3BP), also known as Mac-2-binding protein, is a highly glycosylated secreted protein (Cannarella *et al*. 2020). LGALS3BP has been implicated in the regulation of cell adhesion and immune-related processes. Proteomic profiling of seminal plasma in patients with NOA has demonstrated that LGALS3BP expression may serve as a reliable biomarker for predicting the outcome of TESE procedures (Freour *et al.* 2013). Comprehensive proteomic analyses of seminal fluid from cohorts with NOA might facilitate the identification of additional protein biomarkers predictive of sperm retrieval outcomes. Instead of a single-analyte approach, multimarker diagnostic algorithms are increasingly employed to optimize biomarker panels by evaluating seminal plasma proteomics analyzed via state-of-the-art mass spectrometry-based platforms.

Assessment of cell-free nucleic acids and sperm DNA integrity provides valuable biomarker information for predicting reproductive outcomes. In particular, seminal plasma cell-free DNA (cfDNA) concentration (Di Pizio *et al.* 2021, Wu *et al.* 2025), mitochondrial cfDNA, nucleosomal fragments, and sperm DNA fragmentation rates (Cissen *et al.* 2016*a*,*b*) measured using TUNEL, sperm chromatin structure assay (SCSA), Comet, or sperm chromatin dispersion (SCD)-based platforms (Kaltsas *et al.* 2026) offer important insights into apoptosis, defective chromatin packaging, and DNA damage arising from oxidative stress exposure (Muratori *et al.* 2015, Esteves *et al.* 2021). These biomarkers appear to represent important molecular indicators associated with spermatogenic dysfunction and the prediction of sperm retrieval within seminal plasma.

In a metabolic fingerprinting analysis of seminal plasma obtained from patients with NOA, principal component analysis of Raman spectroscopic data revealed a marked oxidative imbalance in TESE-negative patients compared with TESE-positive patients (Gilany *et al.* 2018). Thus, spectroscopic analysis of testicular signals has been identified as a valuable approach for predicting sperm retrieval outcomes in NOA cases. This analysis focuses on the concentrations of choline (Cho), creatine (Cr), and myo-inositol (MI) (Celik *et al.* 2020). Metabolite profiling of seminal plasma, serum, and testicular tissue samples from patients with NOA may contribute to the prediction of sperm retrieval positivity in TESE procedures.

The balance between oxidative stress and antioxidant capacity in seminal plasma critically influences sperm DNA integrity and fertilization potential (Cai *et al.* 2016). Thus, predictive algorithms can integrate the diagnostic value of ROS, total antioxidant capacity, malondialdehyde, 8-hydroxy-2′-deoxyguanosine, glutathione, superoxide dismutase, catalase, glutathione peroxidase, CoQ10, vitamins C and E, thiol-redox metabolites, and redox-sensitive proteins to improve the assessment of male reproductive potential (Ayad *et al.* 2022, Moustakli *et al.* 2024, Zikopoulos *et al.* 2026). A recent study demonstrated that seminal plasma total antioxidant status (TOS) and oxidative stress index (OSI) values were significantly lower in the mTESE sperm-positive group compared with the mTESE sperm-negative group (Kati *et al.* 2022). In addition, the application of machine learning algorithms has facilitated sperm prediction in patients with NOA, as demonstrated by the positive correlation between semen pH and sperm presence (Xiao *et al.* 2025). Both seminal antioxidant status and semen pH may be predictors of sperm presence. The influence of these factors on spermatogenesis may be mediated by oxidative stress and pH dysregulation within the microenvironment of the germinal epithelium.

Taken together, a multimodal framework integrating seminal plasma biomarkers – including metabolomic, proteomic, and DNA integrity data, combined with machine learning-based feature extraction – may enhance the accuracy of sperm prediction models.

### Genetic predictors in seminal plasma

Several studies have also investigated the potential association between alterations in the expression profiles of various genes in seminal plasma and the prediction of sperm retrieval success. In this context, LASSO regression analysis demonstrated that decreased expression of PIWI-interacting RNA (*piR*)*-61927*, which is implicated in the regulation of genes associated with spermatogenic progression, in extracellular vesicles of seminal plasma may serve as a prognostic biomarker for predicting mTESE outcomes in patients with NOA (Chen *et al.* 2021). The tRNA-derived small RNAs, transfer RNA-derived fragment (*tRF*)*-Val-AAC-010* and *tRF-Pro-AGG-003*, detected in seminal plasma, were identified as potential biomarkers for the diagnosis of NOA, with *tRF-Val-AAC-010* additionally demonstrating predictive value for the presence of sperm within the testicular tissue of NOA cases (Han *et al.* 2022). Circular noncoding RNAs have been demonstrated to participate in a broad spectrum of reproductive processes, including spermatogenesis, sperm motility, fertilization, and early embryonic development (La *et al.* 2023). Differential expression patterns of the three circular RNAs, *Homo sapiens* circular RNA (*hsa_circ*)*_0000277*, *hsa_circ_0060394*, and *hsa_circ_0007773*, identified in seminal plasma may be utilized as predictive biomarkers for sperm retrieval success during the mTESE procedure (Ji *et al.* 2021). Collectively, these studies suggest that small RNAs and circular RNAs may be closely associated with the presence of sperm in patients with NOA. Therefore, RNA sequencing analysis may facilitate the comprehensive identification of these noncoding RNAs that could be incorporated into gene panels. In this regard, Xie *et al.* (2020)*.* developed a diagnostic panel comprising testis-specific extracellular vesicular long noncoding RNAs (lncRNAs) detected in seminal plasma. These panels should be further expanded according to the RNA derivatives and tailored in a patient-specific manner based on individual clinical and biochemical phenotypes.

DNA meiotic recombinase 1 (DMC1, also known as LIM15) is a meiosis-specific recombinase involved in homologous recombination during prophase of the first meiotic divisions in primary spermatocytes (Neale & Keeney 2006). In patients diagnosed with NOA, *DMC1* expression levels in ejaculate have been reported to correlate positively with successful sperm retrieval outcomes following surgical procedures (Ibis *et al.* 2022). Successful progression through meiosis appears to be associated with enhanced spermatogenic output.

The ESX homeobox 1 (*ESX1*) gene is an X-linked homeobox gene with germ cell-specific expression. It has been implicated in the regulation of cell cycle progression and transcriptional control during spermatogenesis. The detection of this biomarker in seminal fluid may be an effective indicator for predicting the presence of residual foci of spermatogenesis in NOA patients (Pansa *et al.* 2014). On the other hand, expression levels of zinc finger MYND-type containing 15 (*ZMYND15*), transition protein 1 (*TNP1*), and protamine 1 (*PRM1*) were significantly reduced in semen samples from patients with NOA compared with normospermic controls (Hashemi *et al.* 2020). A notable observation was the significantly reduced *TNP1* and *PRM1* mRNA levels in the sperm retrieval-negative group compared with the sperm retrieval-positive group (Hashemi *et al.* 2020). Further investigations on this subject may contribute to identifying additional changes in gene expression and noncoding RNAs involved in distinct stages of spermatogenesis.

### Genetic predictors in testicular samples

Spermatogenesis-associated mRNAs and proteins have been characterized in testicular tissue samples for the purpose of predicting the presence of sperm. Heat shock transcription factor Y-linked 1–1 (*HSFY1-1*) and 1–3 (*HSFY1-3*), basic protein Y-linked 2 (*BPY2-1*), lysine demethylase 5C (*KDM5C2*), RNA binding motif protein X-linked 2 (*RBMX2*), and deleted in azoospermia-like (*DAZL1*) mRNAs are known to contribute to male germ cell development and spermatogenic progression (Krausz *et al.* 2015, Ghieh *et al.* 2019). Specifically, these factors are involved in germ cell survival, meiotic regulation, RNA processing, chromatin reprogramming, and spermatogonial differentiation. In patients with NOA exhibiting histopathological evidence of premature maturation arrest, testicular expression levels of specific transcripts, including *HSFY1-1*, *HSFY1-3, BPY2-1*, *KDM5C2*, *RBMX2*, and *DAZL1*, have been proposed as potential predictors of intratesticular sperm presence (Ahmadi Rastegar *et al.* 2015).

The boule-like RNA binding protein (BOLL), also known as BOULE, is a member of the DAZ gene family that plays a critical role in germ cell development, and its deficiency has been associated with azoospermia (Xu *et al.* 2001). As demonstrated by Dai *et al.* (2022), testicular tissues from NOA patients with successful sperm retrieval exhibited higher levels of *BOLL* cell-free mRNA than those from the sperm retrieval failure group (Dai *et al.* 2022). Furthermore, *BOLL* mRNA expression was significantly elevated in the late maturation arrest and hypospermatogenesis groups compared with the early maturation arrest group (Dai *et al.* 2022). Whether the protein expression levels of *ZPBP2* and *BOLL* are closely associated with sperm retrieval success remains an important question. It is essential that the expression of these genes, which are critical for the proper progression of spermatogenesis, is tightly regulated in a temporally and spatially controlled manner within spermatogenic cells.

In the context of spermatogenesis-associated proteins, glial cell line-derived neurotrophic factor (GDNF) plays a critical role in the regulation and maintenance of spermatogonial stem cells within the testicular microenvironment (Meng *et al.* 2000). GDNF is predominantly secreted by Sertoli cells and peritubular myoid cells (Hofmann 2008). In patients with NOA and SCOS, the absence of immunostaining for GDNF in testicular biopsy specimens, together with strong Sertoli cell expression of stem cell factor (SCF), may be associated with a higher likelihood of successful sperm retrieval (Fujita *et al.* 2005).

Testis-specific chromodomain protein Y 1 (CDY1) is a histone acetyltransferase implicated in the replacement of histones by transition proteins and protamines during spermiogenesis. Its protein expression level was reduced in the sperm retrieval-negative NOA group compared with the sperm retrieval-positive group (Heydarian *et al.* 2016). The lysine demethylase 3A (KDM3A)-to-PRM1 ratio may be a robust biomarker for predicting the presence of sperm in patients with NOA undergoing TESE (Javadirad *et al.* 2016). Jumonji domain-containing 1A (JMJD1A) is a well-characterized epigenetic regulator that plays a critical role in the differentiation of round spermatids into mature sperm during spermiogenesis (Pedersen & Helin 2010). A significant upregulation of *JMJD1A* gene expression in testicular biopsy samples was observed in sperm retrieval-positive patients with NOA compared with sperm retrieval-negative patients (Eelaminejad *et al.* 2018).

The ZMYND15 protein functions as a histone deacetylase-dependent transcriptional repressor and plays a critical role in regulating the temporal expression of haploid cell-specific genes during spermiogenesis (Yan *et al.* 2010). TNP1 is involved in chromatin condensation by facilitating the replacement of histones with protamines (PRM1 and PRM2) during spermiogenesis (Rathke *et al.* 2014). Spermatid maturation 1 (SPEM1) participates in cytoplasmic elimination and the subsequent removal of excess cytoplasm from the head and neck regions of developing sperm (Zheng *et al.* 2007). The expression levels of the *ZMYND15* gene and its downstream haploid target genes (*TNP1*, *PRM1*, and *SPEM1*) were lower in sperm retrieval-positive NOA patients undergoing mTESE compared with sperm retrieval-negative patients (Hashemi *et al.* 2018). These studies suggest that epigenetic regulators and their downstream factors are closely associated with the successful progression of spermatogenesis; therefore, their expression levels in testicular tissues may serve as predictive biomarkers for sperm retrieval.

In testicular tissues obtained from patients with NOA, 13 mRNAs were upregulated, whereas 167 miRNAs were downregulated in the unsuccessful sperm retrieval group compared with the successful sperm retrieval group (Fang *et al.* 2019). In addition, 86 testicular miRNAs were undetectable in the unsuccessful sperm retrieval group. Gene Ontology (GO) and Kyoto Encyclopedia of Genes and Genomes (KEGG) pathway enrichment analyses indicated that the differentially expressed miRNAs are involved in key biological processes, including cell apoptosis, proliferation, and differentiation, which are essential for normal spermatogenesis (Fang *et al.* 2019).

The expression of circular RNA monoglyceride lipase (*circ_MGLL*; also known as *circ_0008045*) in testicular tissue was inversely associated with sperm retrieval outcomes in patients with NOA (Shi *et al.* 2022*a*). Given its role in regulating Sertoli cell proliferation and apoptosis, altered circ_MGLL expression may affect spermatogenesis by modulating Sertoli cell abundance and function (Shi *et al.* 2022*a*,*b*). PIWI-interacting RNAs (piRNAs) have been demonstrated to play pivotal roles in spermatogenic progression and are expressed across nearly all testicular germ cell populations. The principal function of these noncoding RNAs, together with their interacting PIWI proteins, is to regulate transposable element activity and modulate their gene expression. In testicular biopsy specimens, Piryaei *et al.* (2024) identified 1.328 differentially expressed piRNAs in idiopathic NOA cases diagnosed with SCOS. Among these, 1.322 piRNAs, including *hsa_piR_012134* and *hsa_piR_020485*, were downregulated, whereas six piRNAs (*hsa_piR_016659*, *hsa_piR_009295*, *hsa_piR_016658*, *hsa_piR_004152*, *hsa_piR_016970*, and *hsa_piR_020485*) were upregulated compared with OA cases. Given the established involvement of different mRNAs, miRNAs, circular RNAs, and piRNAs in spermatogenesis, their altered expression in testicular samples may emerge as a potential biomarker for predicting the presence of sperm in men with NOA.

The coiled-coil, myosin-like BCL2-interacting protein (Beclin-1, also known as BCN1) is associated with testicular injury, with elevated levels considered indicative of testicular damage (Jaiyeoba-Ojigho *et al.* 2025). In testicular tissue, *BCN1* gene expression was significantly elevated in NOA cases with SCOS compared with NOA cases exhibiting hypospermatogenesis and maturation arrest (Zhou *et al.* 2021). A logistic regression model was applied to evaluate the association between *BCN1* expression and sperm retrieval outcomes in mTESE procedures (Zhou *et al.* 2021). Increased *BCN1* mRNA levels were negatively associated with sperm retrieval outcomes. Further studies are warranted to validate these findings and to further characterize alterations in gene expression associated with testicular injury.

Phosphoglycerate kinase 2 (PGK2) is a testis-specific intronless gene predominantly expressed in primary spermatocytes and spermatids, and its absence has been associated with impaired male fertility and diminished sperm motility (Danshina *et al.* 2010). The primary function of PGK2 is to catalyze the reversible conversion of 1,3-bisphosphoglycerate to 3-phosphoglycerate during the late stages of spermatogenesis (Danshina *et al.* 2010). The serine proteinase acrosin (ACR) is localized within the acrosomal region of mature sperm (Adham *et al.* 1997). This enzyme is synthesized and stored within the acrosome as an inactive zymogen precursor termed proacrosin under physiological conditions. Upon activation, acrosin facilitates the lysis of the zona pellucida and promotes sperm penetration through the extracellular matrix surrounding the oocyte. As reported by Ghanami Gashti *et al.* (2023)*,* NOA patients with successful sperm retrieval following mTESE exhibited significantly higher levels of PGK2 and ACR than patients who had negative sperm retrieval outcomes. These associations may be explained by the critical roles of these proteins in the generation and functional maturation of sperm cells.

The zona pellucida binding protein 2 (ZPBP2) is a secreted glycoprotein predominantly expressed by pachytene spermatocytes and spermatids (Tumova *et al.* 2021). It is involved in mediating gamete interaction during the fertilization process (Katoh & Katoh 2003). The expression levels of the testis-specific genes *ZPBP2* and *PGK2* in testicular biopsy specimens were significantly elevated in the sperm retrieval-positive NOA group compared with the sperm retrieval-negative NOA group (Ghanami Gashti *et al.* 2021).

As demonstrated in the aforementioned studies conducted on testicular tissue samples, assessment of the expression profiles of genes and proteins implicated in spermatogenic progression and related cellular processes represents a valuable approach for predicting the presence of sperm in TESE samples from patients with NOA (Fig. 1). The integration of these biomarkers into a gene/proteomic panel may facilitate their broader application across NOA patient populations from diverse ethnic backgrounds.

## Artificial intelligence-based approaches

In recent years, conventional sperm prediction methods in men with NOA have shifted toward machine learning and artificial intelligence applications using multidimensional data (Fontana *et al.* 2024). These analyses contribute to increasing the successful prediction of sperm retrieval during TESE and thus support counseling infertile men, decision-making regarding surgical methods, and individualized treatment.

Advances in supervised machine learning algorithms, including logistic regression, random forest, support vector machines, extreme gradient boosting (XGBoost), and light gradient boosting machines, have facilitated the evaluation of complex, nonlinear interactions among endocrine profiles, clinical parameters, imaging findings, and genetic factors (Kandil *et al.* 2026). A previous study demonstrated that integrative machine learning models combining clinical data (FSH, LH, testosterone, semen volume, age, BMI, ethnicity, and testicular size) achieved an area under the receiver operating characteristic curve (AUC) of approximately 0.80 for predicting sperm retrieval in TESE samples from men with NOA (Zeadna *et al.* 2020). In a multicenter cohort of 2,800 men with NOA who underwent mTESE, an XGBoost machine learning model achieved a mean AUC of 0.91, with robust performance upon validation, yielding AUCs of 0.84 and 0.83 in internal and external cohorts, respectively (Xi *et al.* 2026).

More sophisticated predictive models integrating multiple clinical and laboratory parameters – including FSH, LH, testosterone, inhibin B, testicular volume, history of cryptorchidism, varicocele, genetic variations, and histopathological findings – facilitate individualized estimation of sperm retrieval probability through web-based calculators and nomogram-based interfaces (Xiao *et al.* 2025, Zhou *et al.* 2025, Russo *et al.* 2026). This approach may help avoid unnecessary invasive procedures in men with NOA who have a very low likelihood of successful sperm retrieval. Recent evidence has highlighted the emerging role of multimodal artificial intelligence platforms integrating histopathology, radiomics, and advanced image-based analyses (Jamalirad *et al.* 2025). By jointly interrogating testicular histology, seminiferous tubular morphology, and ultrasound-derived histologic surrogates, these models may improve localization of focal spermatogenic foci beyond the discriminatory capacity of classical clinical predictors.

Despite the substantial advances achieved in this field, several important limitations still remain. Most artificial intelligence models are based on retrospective datasets, are derived from single-center or geographically restricted populations, and are inherently vulnerable to selection bias, missing data, and insufficient external validation. Moreover, the lack of standardized inclusion criteria, heterogeneity in sperm retrieval surgical techniques, and intercenter variability in embryology laboratory workflows may further compromise model generalizability across institutions.

Taken together, artificial intelligence-based sperm retrieval prediction in men with NOA represents one of the most rapidly evolving areas in reproductive biology. The integration of clinical endocrinology, genetic profiling, imaging, histopathology, and machine learning algorithms appears to enhance sperm retrieval prediction and optimize patient selection for TESE applications, thereby potentially reducing the number of unnecessary surgical interventions.

## Possibility of sperm production in NOA patients

For a proportion of men with NOA in whom endogenous sperm production is absent, the development of novel reproductive technologies designed to induce spermatogenesis and produce functionally competent sperm remains a major clinical objective. In an effort to overcome this limitation in the current literature, investigators have utilized diverse methodological strategies, which are outlined in the following section (Fig. 2 and Table 1).

**Figure 2:**
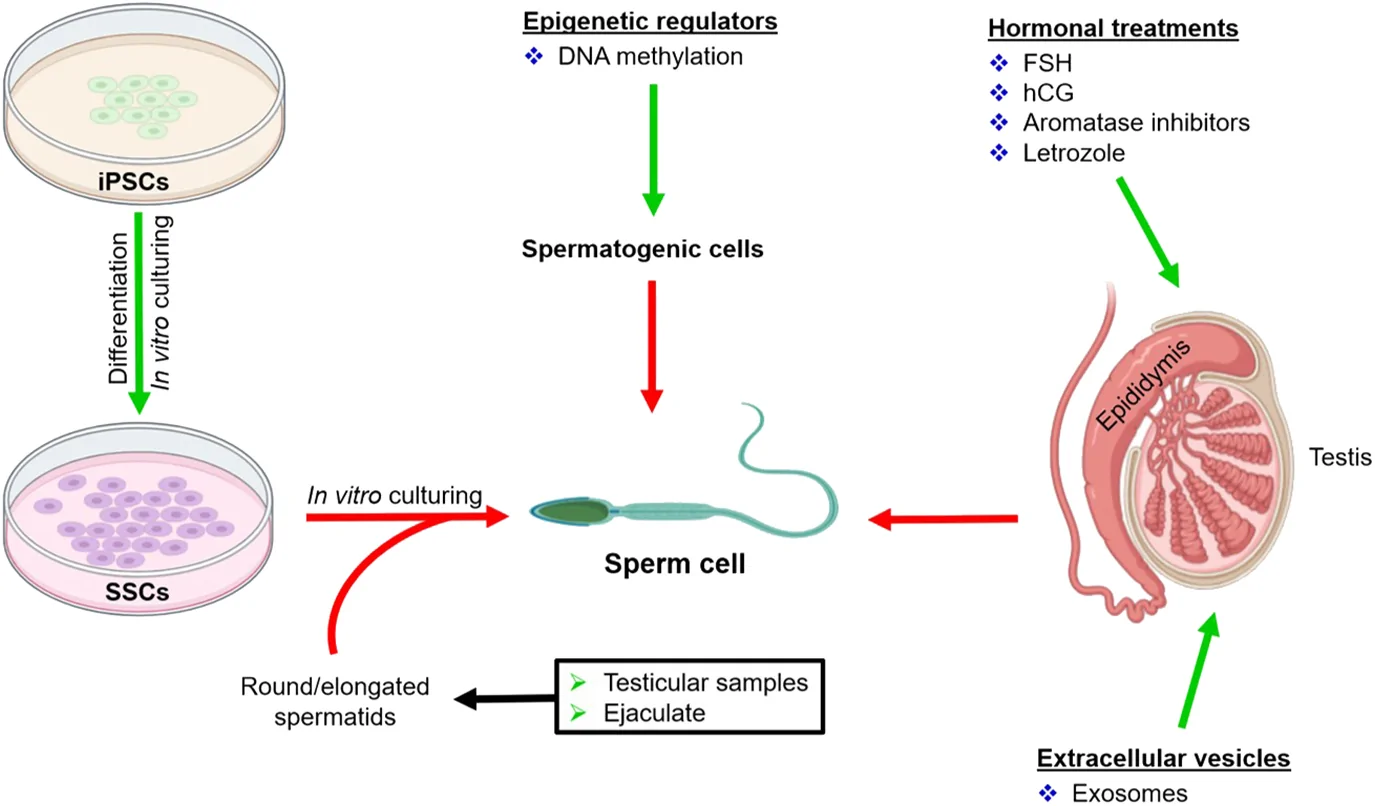
Possible ways of producing sperm cells in NOA patients. *In vitro* culturing of spermatogonial stem cells obtained from testicular samples or from induced pluripotent stem cells (iPSCs) seems to be an option for producing competent sperm cells. Possible approaches include *in vitro* culturing of round or elongated spermatids, epigenetic regulation (especially DNA methylation), hormonal treatments, and applications of extracellular vesicles such as exosomes. FSH, follicle-stimulating hormone; hCG, human chorionic gonadotropin. This figure was created using the BioRender program (BioRender, Toronto, Canada).

### Differentiation of spermatogonial stem cells and round spermatids

Despite severe spermatogenic impairment, spermatogonial stem cells (SSCs) can be recovered from the testicular samples of some men with NOA. In a laboratory setting, these cells can be cultured and directed toward germ cell differentiation, yielding specialized cell populations with developmental potential (Lim *et al.* 2010). Therefore, the culture and expansion of SSCs represent a promising strategy for the treatment of male infertility and provide a valuable platform for investigating reproductive biology (Lim *et al.* 2010). Moreover, the directed differentiation of induced pluripotent stem cells (iPSCs) into sperm through the generation of SSCs offers a promising avenue for producing male gametes in patients affected by NOA (Yao *et al.* 2011). Importantly, sperm production using these methodologies may influence epigenetic reprogramming processes, with particular implications for the establishment and maintenance of DNA methylation (El Hajj & Haaf 2013, Mani *et al.* 2020) of the produced cells. Therefore, extensive investigation of the long-term implications of these technologies is required to ensure their safety and to mitigate potential risks to future offspring.

In a pioneering study, Cremades *et al.* (1999) reported that round spermatids derived from NOA patients showing maturation arrest progressed through spermatogenic differentiation, giving rise to elongating and ultimately elongated spermatids during *in vitro* culture (Cremades *et al.* 1999). Subsequently, a limited number of sperm were generated from these elongated spermatids. However, it is important to emphasize that these experimental approaches provide a foundation for the development of novel therapeutic strategies aimed at generating sperm for patients with NOA exhibiting round spermatid arrest or even primary spermatocyte arrest. Further preclinical and clinical studies are needed to substantiate the feasibility and effectiveness of this strategy for the routine treatment of these patients.

### Endocrine modulation

Treatment with recombinant FSH was associated with an increased sperm retrieval rate in men with SCOS, maturation arrest, and hypospermatogenesis compared with untreated controls (Aydos *et al.* 2003). These results indicate that pure FSH administration may contribute to improved sperm retrieval rates in patients with NOA. Given the pivotal role of testosterone in the regulation of spermatogenesis, reduced testosterone levels may compromise spermatogenic function. Consistent with this notion, a decrease of more than 75% in intratesticular testosterone has been shown to impair sperm maturation in rodent models (Zirkin *et al.* 1989). By activating LH receptors on Leydig cells, hCG promotes testosterone synthesis, with particularly pronounced effects observed in infertile men with hypogonadism (Fraietta *et al.* 2013, Laursen *et al.* 2022). Notably, pre-TESE medical treatment may be unnecessary in patients with NOA when peripheral serum total testosterone concentrations are within the physiological range (Cayan & Yaman 2021).

A proportion of patients with NOA demonstrate elevated aromatase activity, characterized by decreased serum testosterone and increased estradiol concentrations (Schlegel 2012). Accordingly, the use of aromatase inhibitors, including testolactone, anastrozole, and letrozole, may be associated with increased testosterone synthesis and a consequent rise in serum testosterone concentrations. The administration of aromatase inhibitors in men with NOA contributed to restoring spermatogenic activity sufficiently to permit the detection of sperm in the ejaculate (Schlegel 2012). Shiraishi & Matsuyama (2017) reported that therapeutic strategies aimed at increasing testosterone levels, including clomiphene citrate, aromatase inhibitors, and hCG/FSH administration, may improve spermatogenic function in men diagnosed with NOA (Shiraishi & Matsuyama 2017). In patients with NOA exhibiting either SCOS or maturation arrest, letrozole treatment (an estrogen inhibitor) was associated with the emergence of motile sperm, as demonstrated by TESE findings (Kyrou *et al.* 2014). Thus, endocrine modulation through the administration of FSH, hCG, aromatase inhibitors, or estrogen antagonists may be a potential therapeutic strategy for inducing sperm production in patients with hormone levels deviating from normal physiological ranges.

### Epigenetic regulation

Testicular tissues from patients with NOA demonstrated significant hypermethylation of the *SOX30* promoter, resulting in transcriptional inactivation and reduced gene expression (Han *et al.* 2020). Deletion of the *SOX30* gene in mouse models resulted in severe spermatogenic defects characterized by the absence of sperm and consequent male infertility. These findings suggest that SOX30 is indispensable for normal spermatogenesis and that epigenetic silencing of the *SOX30* gene through promoter hypermethylation may contribute to the pathogenesis of NOA. Restoration of *SOX30* expression rescued normal spermatogenesis and consequently restored fertility. Microinjection- and electroporation-based strategies have been proposed as potential therapeutic modalities for restoring spermatogenic function in cases characterized by deficient protein expression, thereby alleviating spermatogenic arrest. This study suggests that epigenetic regulation, particularly DNA methylation in spermatogenic cells, may contribute to the restoration of normal methylation patterns in spermatogenesis-related genes (Fig. 2). Further studies are warranted to determine the potential impact of other epigenetic regulatory mechanisms, including histone modifications, and their direct or indirect interactions with DNA methylation on sperm retrieval outcomes in men with NOA.

### Exosome transfer

Current research efforts are increasingly focused on elucidating the functional properties of extracellular vesicles and their relevance to reproductive biology and male infertility (Wang *et al.* 2025, Parvin *et al.* 2024*a*,*b*). Injection of extracellular vesicles, including exosomes, into busulfan-induced rat models of NOA has been shown to promote the recovery of spermatogenesis (Mobarak *et al.* 2021). Compared with control rats, the restoration of spermatogenesis was evidenced by histopathological staining, spermiogram findings, and hormonal analyses. Furthermore, sperm concentration, motility, and normal morphology in exosome-treated rats were comparable to those observed in control animals (Mobarak *et al.* 2021). In the absence of clinical studies in humans, the use of exosomes may represent a potential strategy for inducing spermatogenesis and facilitating sperm production, warranting further investigation.

## Conclusion and future remarks

Given the restricted spermatogenic potential observed in many patients with NOA, predictive models can offer clinically meaningful insights into their reproductive prospects. Numerous studies have evaluated and validated prediction models incorporating demographic variables, histopathological assessments, serum hormone measurements, seminal plasma biomarkers, and expression profiling. These analytical approaches can be utilized to predict the presence of sperm in patients with NOA (Table 1). Combining carefully selected predictive biomarkers into a multivariable panel and analyzing them using machine learning algorithms may substantially improve the performance and accuracy of sperm prediction in men with NOA.

Over the past decades, significant research attention has been directed toward the development of therapeutic approaches for sperm generation in patients with NOA. Collectively, these findings – encompassing the differentiation of SSCs and iPSCs into sperm cells, hormone-based interventions targeting specific deficiencies, and mechanisms involving DNA methylation and extracellular vesicle transport – hold considerable promise for future therapeutic development. These advances suggest that the induction of sperm production in patients with NOA may become increasingly achievable in the future.

## Declaration of interest

The author declares that there is no conflict of interest that could be perceived as prejudicing the impartiality of the work reported.

## Funding

This research did not receive any specific grant from any funding agency in the public, commercial, or not-for-profit sector.

## Author contribution statement

SO wrote the manuscript and prepared all figures and tables.

## Data availability

The datasets generated during the current study are available from the corresponding author on request.
